# The Effect of Judgement Bias on Cue Utilization for Shot Prediction in Basketball Athletes

**DOI:** 10.3390/brainsci11081058

**Published:** 2021-08-11

**Authors:** Yawei Li, Tian Feng

**Affiliations:** 1 Department of Sports, Physical Education College of Zhengzhou University, Zhengzhou 450044, China; yaweili@vip.126.com; 2 Department of Physical Education, Physical Education College of Zhengzhou University, Zhengzhou 450044, China

**Keywords:** shot prediction, judgement bias, cue utilization, basketball

## Abstract

Background: Concerning the judgments bias and cue utilization in basketball athletes, previous shot anticipation tasks were hard to examine in regards to whether the experts’ judgement bias relies more on the cue of the player’s body or the ball trajectory. Methods: Four types of body–ball cues shots were employed: IN–IN, IN–OUT, OUT–IN, and OUT–OUT. Four temporal stages (i.e., shooting, rising, high point, and falling) were divided during a shot. Forty-two participants predicted the fate of the ball after watching the shot videos. Results: The results suggested that for the shooting, rising, and high point phase, compared to the non-athletes, the experts provided superior predictions for IN–IN condition and OUT–IN condition but fewer accurate predictions for IN–OUT condition and OUT–OUT condition. Moreover, a higher bias toward predicting the shots as “in” for the athletes than the non-athletes under early temporal conditions was confirmed. Conclusions: These findings strengthen the idea that the IN cues from both body information and ball trajectory could elicit the experts’ judgement bias for made shots and then influence their response, thus rendered two distinct (e.g., impeding and facilitating) effects for the incongruent body–ball cues, respectively.

## 1. Introduction

Action anticipation is the ability to predict an event’s outcome [1]. In the context of basketball, keeping alert and making accurate anticipation of every shot can help a team win. Therefore, seeking the cognitive process of action anticipation of a shot is essential for basketball players.

As noted by several studies, expert basketball players demonstrated better anticipation than novices [2,3,4], but the advantage was affected by the shot results [5,6,7]. Interestingly, experts could not predict better in all basketball shots. Studies have found that the advantage of athletes only exists in predicting successful shots. Cañal-Bruland, Balch, and Niesert [7] found higher accuracy for basketball players when they were predicting their own made shots. Moreover, another study demonstrated that, compared to the novices, expert players’ predictions were even less accurate for their own missed shots [5]. That is, a judgement bias towards better prediction of their successful shots was illustrated when the athletes were performing basketball shots. Moreover, the results above were confirmed by signal-detection theory.

The experts’ judgement bias has been interpreted as the effect of “regulatory fit” [6]. The theory of regulatory focus presents two types of individual focus, namely promotion focus and prevention focus [8]. People who have a promotion focus adopt more positive behavioral strategies to win the game. On the contrary, individuals with a prevention focus are more inclined to avoid failure and adopt conservative strategies [9]. The effect of regulatory fit emerges when the action strategy is consistent with one’s focus. Therefore, predicting a shot as “in” is a positive strategy that may fit experts’ promotion focus, causing a regulatory fit. Since expert basketball players have higher motivation to win a match, they show a self-regulatory focus, i.e., “focus on gains”, and a behavioral strategy, i.e., “search for a win”. A study found that the regulatory fit led to a broader scope of attention in a basketball shot task [10]. As explained earlier, experts with a “focus on gains” were sensitive to positive results [11], so the regulatory fit could help them improve their performance. Another possible explanation is that the athletes’ judgment bias might be caused by top-down processing. It is demonstrated that physical movement and motor imagery share a common process [12], and action prediction is regarded as “motor resonance”, which reflects a mirror-like activity in the observer’s motor system during action observation. Though this process has always been identified as an inner, automatic replica of the observed movement, predictive coding models suggest that top-down modulation makes motor resonance a less faithful replica [13]. Therefore, considering the notion that mirroring the observed actions of others underlies action understanding, basketball experts’ judgement bias was explained.

There are two kinds of shot prediction, that is, predicting one’s own shot and predicting another’s shot. Cañal-Bruland, Balch, and Niesert [7] asked basketball players to perform free shots. When the ball was released by themselves or others, basketball players’ and observers’ vision was occluded, and they were asked to report the fate of the ball. Results showed higher accuracy for basketball players when they were observing their own made shots. Therefore, with the accumulation of professional training, athletes can compare their proprioception with the standard action. By doing this, experts can be aware of their movement errors. On the other hand, successfully predicting another’s shot as an observer maybe even more important than anticipating one’s own shooting. As a player, watching others throw a ball during a basketball game occurs much more often than throwing it oneself. However, previous studies revealed contradictory results. Our research showed collegiate players and recreational players demonstrated higher accuracy than the non-athletes when predicting another’s shooting. This kind of advantage may be benefited from the process of embodied cognition, which suggests that cognitive processes are deeply rooted in the interactions between the body and the environment [14]. However, in the study of Cañal-Bruland, Balch, and Niesert [7], basketball observers did not predict more accurately for the made shots as the players did. To explain this, the motivational differences and the quantity and quality of information must be discussed.

It is worth noting that the cues of a successful shot come not only from the shot result of the ball but also from the shooter’s body. In general, a player’s body posture shows his or her early action intention, and the ball trajectory shows a later shot result. However, a recent study found the collegiate players were superior to the non-athletes in predicting made shots regardless of whether they watched the shooting, rising, high point, or falling phase of a shooting [6]. However, the kind of cues that induce an experts’ judgement bias remains unclear. A few researchers surveyed how the movement cues are applied by experts in different temporal phases and shot results. In a study of table tennis, Zhao et al. [15] created congruent and incongruent body–ball cues and found players were superior in anticipating the ball trajectory using body movements’ incongruent conditions, but the superiority vanished when the ball trajectory was incongruent to the action. Nevertheless, before a racquet player starts a serve, he or she has already decided the ball direction. The ball trajectory is always congruent with their action trend. To some extent, employing inauthentic video clips in their study was questionable. Previous research of shot anticipation was hard to identify in regards to whether the experts rely more on the body movement of the players or the ball trajectory.

During an anticipation task, the interaction between temporal information and two kinds of cues is also crucial. Using the temporal occlusion paradigm, Wu et al. [3] divided the temporal course of a shot (which had 11 continuous pictures) into early (three pictures), middle (six pictures), and late phases (nine pictures), and asked basketball players to predict the result under different temporal phases. It was revealed that experts presented higher accuracy than the novices when watching three or six pictures. That is, the experts can make better use of incomplete cues than novices. Concerning the judgement bias, Li and Feng [6] compared the accuracy of basketball players with varying levels and non-athletes under different temporal conditions. It was reported that collegiate players outperformed recreational players and non-athletes regardless of the temporal condition when presented with successful shots, supporting the notion that experts can match the proprioception of their shots to the visual information of others’ shots. In terms of the “regulatory fit” theory, athletes’ improvement is supposed to have higher motivation to exert their best effort to win a match, reflecting a regulatory focus of promotion. Additionally, this finding was also reported by studies investigating table tennis and tennis. A study by Zhao, Lu, Jaquess, and Zhou [15] showed that, compared to middle-level and novice players, expert athletes used early, effective information to get better predictions. Moreover, research on squash indicated that the most critical periods for extracting information about stroke direction are 160–80 ms before racket–ball contact and the ball flight, arising at least 80 ms after contact [16]. A possible explanation for this might be that athletes have a better ability of visual perception, which is gained in years of sports training. Generally speaking, early visual information includes body cues, and later visual information involves the flying trajectory of the ball. Regarding the judgement bias, whether it affects the early or later cue utilization and the performance of shot prediction in basketball athletes has not been surveyed by researchers.

A basketball player always wants to hit the ball, though the result may sometimes be disappointing. Athletes can never know the result of a shot until the ball goes into the basket or bounces away, that is, his or her action of shot may be incongruent with the actual ball trajectory. Concerning this, the present study was designed to determine the interplay of kinematic information and ball trajectory during a shot prediction as well as the effect of judgement bias and temporal phase. We used four types of expectation–result (body–ball) shots: IN–IN, IN–OUT, OUT–IN, and OUT–OUT. As was pointed out earlier, experts have judgement bias for made shots, so we predicted that they will outperform the novices in IN–IN condition but perform worse in OUT–OUT condition (Hypothesis 1). As far as the cue utilization is concerned, it is hypothesized that experts will present lower accuracy in IN–OUT condition than the non-athletes if their judgement bias relies on body cues; on the contrary, if the experts’ judgement bias relies on the ball cues, they will show better performance in OUT–IN condition (Hypothesis 2). Moreover, according to previous studies [5,7], signal-detection theory was employed to examine the judgement bias. We hypothesized that athletes were significantly more biased toward predicting the shots as “in” than the non-athletes (Hypothesis 3).

## 2. Materials and Methods

### 2.1. Participants

We recruited 42 male subjects in this experiment. They were 22 collegiate players (age: 20.91 ± 0.87 years) and 20 non-athletes (age: 20.90 ± 0.97 years). The collegiate athletes were on a college basketball, and they practiced 9.11 ± 2.90 h/week. The non-athletes were university students, and they never took part in sports training. Two groups were of similar age: *t* (40) = 0.032, *p* = 0.975, *d* = 0.011. Notably, power analysis of R-M ANOVA design was conducted with GPower software [17], using the setting for expected effects size at 0.25, α-level at 0.05, sample size at 42, and the power (1-β) was 0.89. Informed consent, including the purpose, methods, obligations, responsibilities, and rights of the participants, was obtained before we started the study. The Ethical Committee of Physical Education College of Zhengzhou University ethically approved the experiment (No. 2019002), and informed consent has been given by the subjects.

### 2.2. Materials

Two professional right-handed, male basketball players shot free throws after warming up, and a digital camera (Canon EOS 5D Mark IV, focal length of 3.5 mm) recorded their shot with a speed of 60 frames/second. The height of the camera was 1.70 m. The players deliberately performed 60 made and missed shots. For made ones, players were asked to try their best to shoot successful balls. If the player succeeded, the shot would be marked as “IN–IN”, that is to say, an “IN” body posture and an “IN” ball trajectory. Otherwise, the shot would be marked as “IN–OUT”, which represented an “IN” body posture and an “OUT” ball trajectory. For missed ones, players were asked to shoot missed balls, but the ball must touch the hoop. If the ball successfully bounced off, the shot would be marked as “OUT–OUT”, which is an “OUT” body posture and an “OUT” ball trajectory. If the ball went into the basket, the shot would be marked as “OUT–IN”, which represented an “OUT” body posture and an “IN” ball trajectory. Therefore, four types of body–ball cues were divided: (1) IN–IN: the player is required to perform an IN shot, and he does it; (2) IN–OUT: the player is required to perform an IN shot, but the ball is OUT; (3) OUT–IN: the player is required to perform an OUT shot with hitting the basket, but the ball is IN; and (4) OUT–OUT: the player is required to perform an OUT shot hitting the basket, and he does it. Each type had ten shots. The period of the video was from when the player held the ball to when the ball hit or missed the basket. Each video was about 1500 ms, and 40 videos of free throws were chosen.

**Figure 1:**
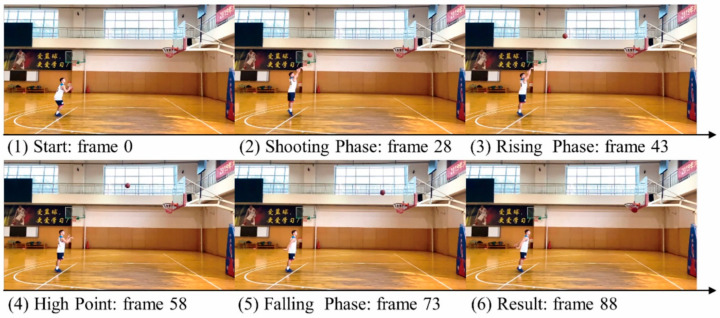
Example of the experimental stimuli. Written informed consent was obtained for the publication of this image.

In addition, concerning both the temporal information and previous research results, each shot was divided into the four temporal conditions: (1) the shooting stage (467 ms), (2) the rising stage (717 ms), (3) the high point (967 ms), and (4) the falling stage (1217 ms). To prevent the participants from seeing the results, we excluded the last 283 ms. Table 1 shows the characteristics of each temporal condition (the number of frames, presentation time, and ball position). A total of 160 video clips (40 video clips × 4 temporal conditions) were used in the present prediction experiment. In the shot-prediction task, subjects observed the shooting, rising, high point, and falling phase of a free shot and anticipated the ball’s fate. Figure 1 presents an example of the experimental stimuli.

### 2.3. Experimental Procedure

**Figure 2:**
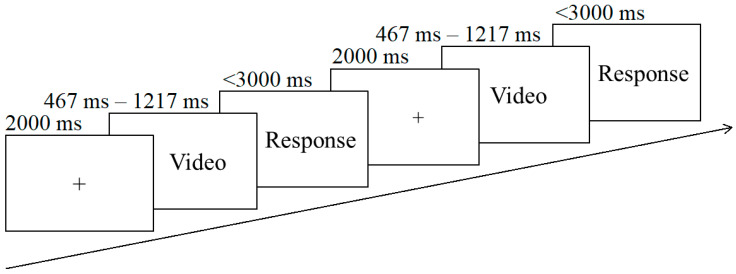
Example of trials.

The study was conducted in a quiet room at the Physical Education College of Zhengzhou University, and each subject was individually tested. A questionnaire referring to their individual information was given to the participants. Next, they took part in an action-prediction task. The subjects were seated in front of a 23.8-inch screen, and the distance was 60 cm in the task. A two-alternative, forced-choice task was performed, requiring participants to predict the result of the free shot. Firstly, a cross-shaped fixation was showed on the screen for 2000 ms. Secondly, a free shot video was played at a resolution of 1088 × 608 pixels. After watching each video, the subjects anticipated the ball’s fate (“F” key for made balls and “J” key for missed balls) as quickly and accurately as they could. Answers more than 3000 ms after the stimulus display were considered wrong. The next trial started after the response had been made or after the maximum time elapsed. Subsequent trials commenced immediately after the previous trial (Figure 2). All participants completed 15 practice trials with feedback after they read the task instructions. 10 IN–IN shots, 10 IN–OUT shots, 10 OUT–IN shots, and 10 OUT–OUT shots (40 shots) were used and arranged into 160 experimental trials, which were 40 shots × 4 temporal conditions. There were eight random blocks, and each had 20 shots for one condition. The experiment was performed with the software E-prime 2.0 (Psychology Software Tools, Sharpsburg, PA, USA). The response keys were counterbalanced across all subjects. The formal experimental trials provided no feedback and a 30-s break between each block. The entire experiment lasted approximately 50 min. 

### 2.4. Statistical Analyses

Assessing the reaction time of the participants was difficult because the participants were able to make their decisions before the option to press the key became available, and each prediction was made after the picture disappeared. Therefore, only the accuracy results were evaluated. Normal distributions for the accuracies in two groups under each temporal condition were confirmed (z < 0.151, *p* > 0.166 in all instances). To test our hypotheses regarding the anticipation in different shot types of body–ball cues and temporal conditions, a three-way RM-ANOVA was conducted with the dependent variable accuracy, the between-subjects variable group, and within-subjects variable shot type of body–ball cues (IN–IN, IN–OUT, OUT–IN, OUT–OUT) and temporal condition (467 ms, 717 ms, 967 ms, and 1217 ms). Additionally, signal-detection theory was used to calculate the perceptual sensitivity (d′) and judgement bias (c) [18]. Sensitivity of accuracy is the “ability of a participant to discriminate between two sets of stimuli (e.g., IN or OUT shots)” [5] and is also considered as the capacity to detect a discrepancy in the kinematic patterns of the different videos [15]. Judgement bias reflects a participant’s likelihood to provide a specific response over the other. Concerning the final fate of the ball and the response of the subjects, the hit rate (correct prediction for IN shots) and the false-alarm rate (incorrect prediction for OUT shots) were calculated converted to sensitivity and judgement bias scores via z-transformation: Sensitivity = z(TP) – z(FN) and judgement bias = −0.5 × (z(TP) + z(FN)). Therefore, two RM-ANOVA were conducted with the dependent variable sensitivity (d’), judgement bias (c), and within-subjects variable temporal condition (467 ms, 717 ms, 967 ms, and 1217 ms). Greenhouse–Geisser correction was applied when the assumption of sphericity was violated, and Bonferroni-corrected post-hoc *t*-tests were used to identify the main effects and interactions.

## 3. Results

**Figure 3:**
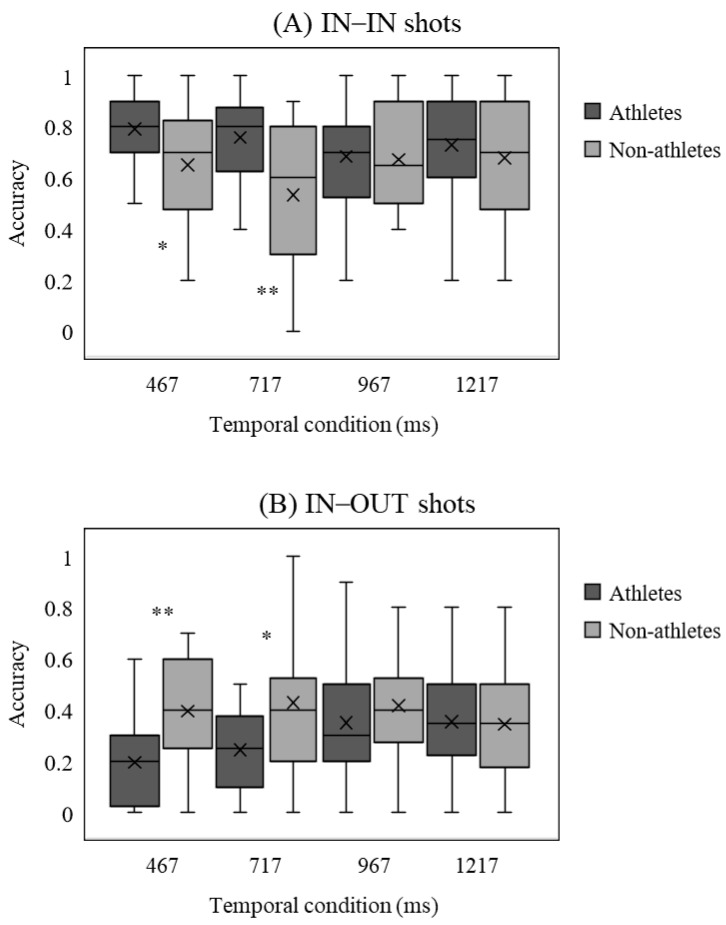

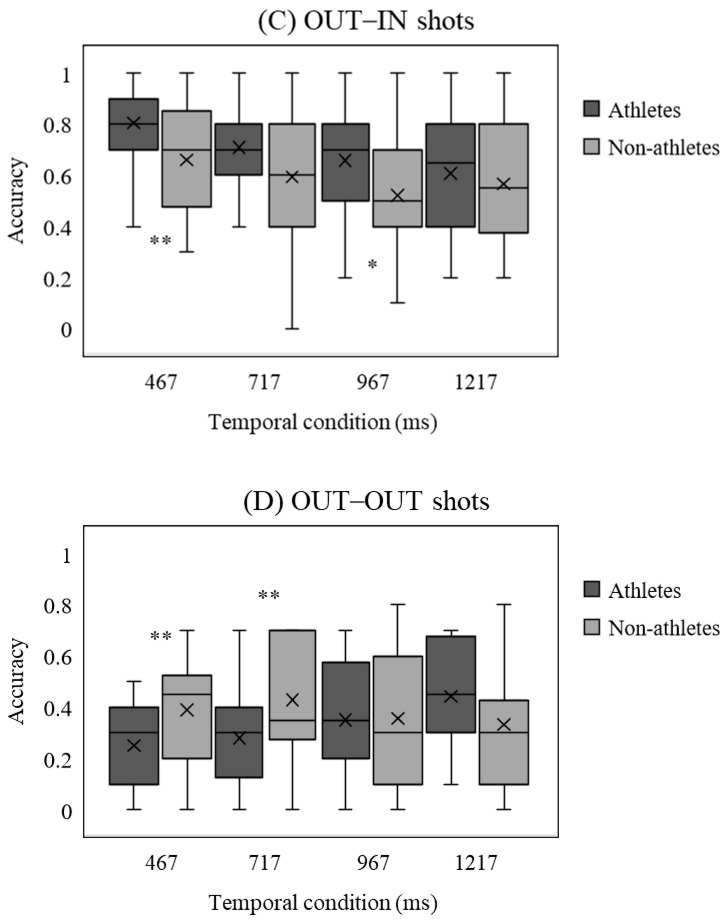
Mean accuracy of every body–ball cue for athletes and non-athletes under all temporal conditions. Bars indicate standard errors. ^∗^ *p* < 0.05, ^∗∗^ *p* < 0.01.

Significant main effects of the shot type of body–ball cues, *F* (3, 120) = 25.64, *p* < 0.001, η_p_^2^ = 0.57, and the interaction between shot type × temporal condition, *F* (9, 360) = 2.69, *p* < 0.05, η_p_^2^ = 0.12, were shown. Additionally, the three-factor interaction between shot type × temporal condition × group was significant, *F* (9, 360) = 2.93, *p* < 0.05, η_p_^2^ = 0.13. Post-hoc analysis of the four shot types and four temporal conditions found that for IN–IN shots, players had higher accuracy than the non-athletes under the shooting (athletes: 0.79 ± 0.15, non-athletes: 0.65 ± 0.25) and rising phases (athletes: 0.76 ± 0.16, non-athletes: 0.53 ± 0.28, *p* < 0.05, *d* > 0.68 in all instances); for OUT–IN shots, better performance of athletes than the non-athletes was found under the shooting (athletes: 0.80 ± 0.14, non-athletes: 0.66 ± 0.24) and high point phase (athletes: 0.66 ± 0.21, non-athletes: 0.52 ± 0.23, *p* < 0.05, *d* > 0.64 in all instances). However, experts showed lower accurate predictions for IN–OUT condition and OUT–OUT condition than the non-athletes (*p* < 0.05, *d* > 0.72 in all instances) for the shooting (IN–OUT: athletes: 0.20 ± 0.17, non-athletes: 0.40 ± 0.24; OUT–OUT: athletes: 0.25 ± 0.16, non-athletes: 0.39 ± 0.21) and rising phases (IN–OUT: athletes: 0.25 ± 0.16, non-athletes: 0.43 ± 0.28; OUT–OUT: athletes: 0.28 ± 0.17, non-athletes: 0.43 ± 0.24, Figure 3.). Moreover, both the athletes and the non-athletes showed higher accuracy for IN-IN and OUT-IN shots than IN-OUT and OUT-OUT shots (*p* < 0.05, *d* > 0.45 in all instances).

**Figure 4:**
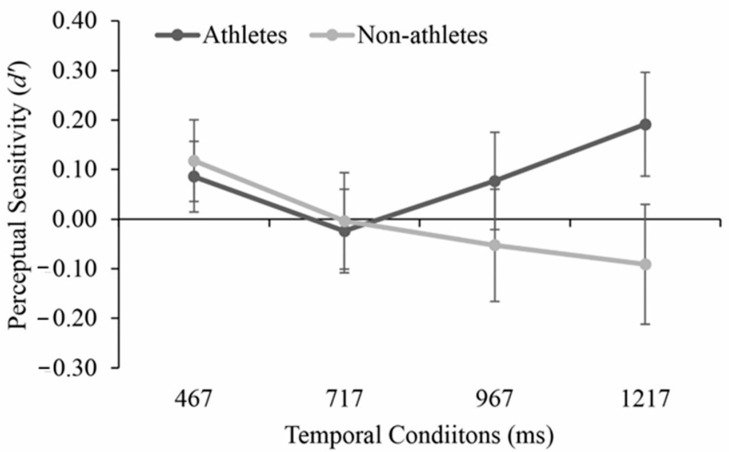
Mean perceptual sensitivity for athletes and non-athletes under four temporal conditions. Bars indicate standard errors.

**Figure 5:**
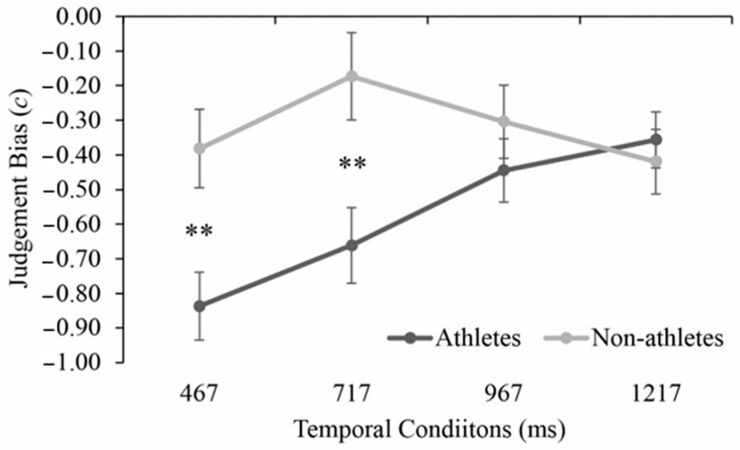
Mean judgement bias (c) scores for athletes and non-athletes under four temporal conditions. Bars indicate standard errors. ^∗∗^ *p* < 0.01.

As illustrated in Figure 4, The ANOVA of sensitivity indicated no significant main effects of the group, *F* (3, 120) = 1.428, *p* = 0.239, η_p_^2^ = 0.034, or temporal condition, *F* (3, 120) = 0.563, *p* = 0.615, η_p_^2^ = 0.014. The interaction between the two factors did not reach significance, *F* (3, 120) = 1.222, *p* = 0.304, η_p_^2^ = 0.030. For the analysis of judgement bias, significant main effects of group and temporal condition as well as their interaction were found, *F* > 4.595, *p* < 0.05, η_p_^2^ > 0.103 in all instances. The post-hoc tests of judgement bias under the four temporal conditions per group revealed that under the condition of 467 ms and 717 ms, the players (467 ms: −0.83 ± 0.20, 717 ms: −0.66 ± 0.26) were significantly more biased toward predicting the shots as “IN” than the non-athletes (467 ms: −0.38 ± 0.28, 717 ms: −0.17 ± 0.31), *p* < 0.01, *d* > 0.71 in all instances (Figure 5). As illustrated in Figure 4, The ANOVA of sensitivity indicated no significant main effects of the group, *F* (3, 120) = 1.428, *p* = 0.239, η_p_^2^ = 0.034, or temporal condition, *F* (3, 120) = 0.563, *p* = 0.615, η_p_^2^ = 0.014. The interaction between the two factors did not reach significance, *F* (3, 120) = 1.222, *p* = 0.304, η_p_^2^ = 0.030). For the analysis of judgement bias, significant main effects of group and temporal condition as well as their interaction were found, *F* > 4.595, *p* < 0.05, η_p_^2^ > 0.103 in all instances. The post-hoc tests of judgement bias under the four temporal conditions per group revealed that under the condition of 467 ms and 717 ms, the players (467 ms: −0.83 ± 0.20, 717 ms: −0.66 ± 0.26) were significantly more biased toward predicting the shots as “IN” than the non-athletes (467 ms: −0.38 ± 0.28, 717 ms: −0.17 ± 0.31), *p* < 0.01, *d* > 0.71 in all instances (Figure 5). 

## 4. Discussion

In the case of the action anticipation of basketball players, our study firstly reported the effect of judgement bias on early and later cue utilization by assessing the interaction of temporal information and body–ball cues. The performances of shot prediction in basketball athletes and novices were recorded, and the results showed that for the shooting, rising, and high point phase, the experts outperformed the non-athletes for IN–IN condition and OUT–IN condition, but athletes made fewer accurate predictions for IN–OUT condition and OUT–OUT condition than the non-athletes. These results were discussed in the context of temporal information, judgement bias, and cue utilization.

Temporal information has been presented to influence the result of action prediction. The presented results confirmed that the advantage of experts’ anticipation emerged in the shooting and rising phases. As suggested by previous research, the advantage of athletes exists in the early and middle temporal stages but diminishes with the increase of temporal information [2,3]. In the case of temporal course, Vicario et al. [19] depicted the action of shot earlier, synchronously, or late to the natural course and asked the subjects to detect the compatibility. Their study found that the athletes only predicted the action more accurately in the early synchronous phase. Thus, the early temporal advantages may be since expert basketball players had better ability of visual perception [3]. Besides, increased neuronal excitability related to motor observation was found when the elites watch the shot videos [2]. Earlier studies on the prediction of racquet sports showed that professional athletes use effectively early cues to accomplish anticipation of the ball direction [15]. Albeit the present result of perceptual sensitivity (d′) did not show group difference, the non-significance was in line with other studies by Cañal-Bruland, Balch, and Niesert [7] and Maglott, Chiasson, and Shull [5], and it may be due to the fact that the experts we chose were collegiate players but not national players.

Regarding the body action and ball trajectory between an IN and an OUT shot, we used four body–ball interactions to test the feature of judgement bias and cue utilization for athletes and non-athletes. The results revealed that the experts performed better than the novices in IN–IN condition, and the results of criteria c confirmed their judgement bias for IN shots as well. In the present experiment, a player’s shooting intention influenced his body cues, and the ball trajectory was congruent with the shot result. Thus, the findings suggested that if the video showed that a player wanted to perform a successful shot (e.g., his body cues are “IN”), and the ball went into the basket (e.g., ball trajectory is “IN”), when the athletes watched the video, he would make a better prediction. An electrophysiological study showed that compared to OUT shots, watching IN shots in the early temporal phase elicited significant activity of frontoparietal action observation network in basketball players but not in novices [3]. These results corroborated our notion that elite players are inclined to concentrate on the IN balls depending on their long-term sportive training as well as their regulatory focus of promotion.

With respect to the OUT shots, early studies revealed a trend that as the skill level increased, the performance of predictions for OUT shot gradually decreased regardless of the temporal conditions [6], which was confirmed by the present study, showing that the players had fewer accurate predictions for OUT–OUT condition than the non-athletes. Some studies pointed out that coping with an OUT shot involves more activities about motion perception, understanding, and intention analysing, which may be reflected in different brain areas [3,20,21]. A TMS study found an increase of corticospinal excitability specific for the hand muscle when the athletes watched OUT shots compared with IN shots, especially for the moment when the ball left the hand [13]. To the best of our knowledge, though basketball players were supposed to have better ability of visual perception [3], experts’ worse performance for prediction of OUT shots may be related to their judgement bias, which further develops into subjective expectations or overconfidence. In line with this, studies have found that sometimes skilled athletes’ subconscious can inhibit objective visual information [22]. Concerning the regulatory focus theory, the progress of mirroring could be affected by other high-level factors, such as expectations, the goal, and the intention of action [23]. This may be explained by the fact that the judgements of professional athletes are influenced by their expectation for scores, goal orientation of triumph, and intention to win, and therefore the experts showed lower accuracy than the non-athletes in the OUT–OUT condition even though they are specially trained for years and as such are familiar with the observed action. 

To answer the question of how players with different levels make predictions in the context of the body–ball interaction, we focused on the incongruent conditions: the IN–OUT and the OUT–IN shots. It was hypothesized that if the experts’ judgement bias relies on the body cues, they will show lower accuracy than the non-athletes in IN–OUT condition; otherwise, athletes’ superiority for OUT–IN condition will be found due to the fact that their judgement bias depends on the ball cues. Intriguingly, both two assumptions were proven. Our results revealed that concerning the IN–OUT shots, the experienced players predicted less accurately for unexpected OUT shots than the non-athletes in the shooting and rising phase. Additionally, they had better performance for OUT–IN shots than the non-athletes in the shooting and high point phase, which means that they had advantages for unexpected IN shots during early and middle temporal stages. These findings demonstrated that the IN cues from both body action and ball trajectory could elicit the experts’ judgement bias for made shots and then influence their response and rendered two distinct (e.g., impeding and facilitating) effects for IN–OUT and OUT–IN shots, respectively. According to Güldenpenning et al. [24], anticipation performance is affected by deceptive actions in sport-related tasks in both novice and expert athletes; however, experts still outperform novices when facing deceptive actions in sport-related tasks. Inconsistently, the paradigm of the present study could be considered as unplanned deceptive actions, which added relevant evidence by proving that the expectation and shot results mediate their capability to distinguish deceptive actions.

However, these results were contradicted by of other previous studies. Aglioti, Cesari, Romani, and Urgesi [2] thought that experts could discriminate between erroneous and correct performance, and some studies demonstrated that only experts could use body movements to make predictions [15]. Compared to expert watchers and novices, elite athletes were found to extract kinematic information from the player’s body movements when watching the player who was holding the ball [2]. In another basketball research, the lower part of the player’s body was considered critical visual information to predict shot success [4]. Moreover, a study on volleyball floating services showed that although observational training could improve the understanding of ball trajectory, only athletes could base their predictions on body kinematics [25]. To test the utilization of kinematic and ball information, Zhao, Lu, Jaquess, and Zhou [15] created congruent and incongruent body–ball cues and, by editing the clips, found that experts’ superiority vanished when the ball trajectory was incongruent to the action. Their results could be a hint that though the experts may be confused when facing incongruent body–ball cues as well as the non-athletes, they did better in the task conditions involving body movement. However, Anderson, et al. [26] claimed that the observer’s visual features must be congruent with the ecological features of the action so that a likely kinematic effect in the environment can be coded with the action in form of appropriate physical space in the right direction. In line with this view, the experts’ advantages may only exist in the genuine sportive situations, so using impossible videos (e.g., changing the original clip of ball trajectory to an opposite one and adding it to the following of a serving clip) is rather noneffective and hard to investigate in regards to athletes’ action-prediction ability. In this point of view, the present study may be the first to examine the shot expectation–result interaction and body–ball cues by using a novel and ecological approach.

## 5. Conclusions

The present study investigated the effect of judgement bias on early and later cue utilization by assessing the interaction of temporal information and body-ball cues in basketball players with different expertise levels. The results demonstrated that for the shooting, rising, and high point phases, experts provided better predictions for IN–IN condition and OUT–IN condition but lower accurate predictions for IN–OUT condition and OUT–OUT condition than the non-athletes. Moreover, athletes demonstrated a higher bias toward predicting their shots as “in” than for the non-athletes under early temporal conditions. These findings demonstrated that the IN cues from both body action and ball trajectory could elicit the experts’ judgement bias for made shots and influence their response, and the results extended the understanding of the role of IN-relevant information in the body–ball cues relation during the early temporal courses in action anticipation.

## Figures and Tables

**Table 1:** Experimental Stimulus Information.

Temporal Condition	Frames	Presentation Time (ms)	Ball Position
(1) Shooting Phase	28	467	The player releases the basketball.
(2) Rising Phase	43	717	The basketball reaches approximately the midpoint between the player’s hand and the high point.
(3) High Point	58	967	The basketball reaches the climax of its trajectory.
(4) Falling Phase	73	1217	The basketball approaches the basket.

## References

[1] Abreu A.M. (2014). Action anticipation in sports: A particular case of expert decision-making. Trends Sport Sci..

[2] Aglioti S.M., Cesari P., Romani M., Urgesi C. (2008). Action anticipation and motor resonance in elite basketball players. Nat. Neurosci..

[3] Wu Y., Zeng Y., Zhang L., Wang S., Wang D., Tan X., Zhu X., Zhang J., Zhang J. (2013). The role of visual perception in action anticipation in basketball athletes. Neuroscience.

[4] Uchida Y., Mizuguchi N., Honda M., Kanosue K. (2014). Prediction of shot success for basketball free throws: Visual search strategy. Eur. J. Sport Sci..

[5] Maglott J.C., Chiasson D., Shull P.B. (2019). Influence of skill level on predicting the success of one’s own basketball free throws. PLoS ONE.

[6] Li Y., Feng T. (2020). The effects of sport expertise and shot results on basketball players’ action anticipation. PLoS ONE.

[7] Cañal-Bruland R., Balch L., Niesert L. (2015). Judgement bias in predicting the success of one’s own basketball free throws but not those of others. Psychol. Res..

[8] Higgins E.T. (2000). Making a good decision: Value from fit. Am. Psychol..

[9] Rupp A., Steinwachs D.M., Salkever D.S. (1985). Hospital payment effects on acute inpatient care for mental disorders. Arch. Gen. Psychiatry.

[10] Memmert D., Unkelbach C., Ganns S. (2010). The Impact of Regulatory Fit on Performance in an Inattentional Blindness Paradigm. J. Gen. Psychol..

[11] Avnet T., Higgins E.T. (2003). Locomotion, assessment, and regulatory fit: Value transfer from “how” to “what”. J. Exp. Soc. Psychol..

[12] Wohlschläger A., Wohlschläger A. (1998). Mental and manual rotation. J. Exp. Psychol. Hum. Percept. Perform..

[13] Finisguerra A., Amoruso L., Urgesi C. (2020). Beyond Automatic Motor Mapping: New Insights into Top-Down Modulations on Action Perception. Modelling Human Motion.

[14] Wilson M. (2002). Six views of embodied cognition. Psychon. Bull. Rev..

[15] Zhao Q., Lu Y., Jaquess K.J., Zhou C. (2018). Utilization of cues in action anticipation in table tennis players. J. Sports Sci..

[16] Abernethy B. (1990). Anticipation in squash: Differences in advance cue utilization between expert and novice players. J. Sports Sci..

[17] Faul F., Erdfelder E., Buchner A., Lang A.-G. (2009). Statistical power analyses using G* Power 3.1: Tests for correlation and regression analyses. Behav. Res. Methods.

[18] Macmillan N.A., Kaplan H.L. (1985). Detection Theory Analysis of Group Data. Estimating Sensitivity From Average Hit and False-Alarm Rates. Psychol. Bull..

[19] Vicario C.M., Makris S., Urgesi C. (2016). Do experts see it in slow motion? Altered timing of action simulation uncovers domain-specific perceptual processing in expert athletes. Psychol. Res..

[20] Julie G., Decety J. (2015). Functional anatomy of execution, mental simulation, observation, and verb generation of actions: A meta-analysis. Hum. Brain Mapp..

[21] Pavlova M., Sokolov A.N., Birbaumer N., Krägeloh-Mann I. (2008). Perception and Understanding of Others’ Actions and Brain Connectivity. J. Cogn. Neuroence.

[22] Vickers J.N. (1996). Visual control when aiming at a far target. J. Exp. Psychology. Hum. Percept. Perform..

[23] Finisguerra A., Maffongelli L., Bassolino M., Jacono M., Pozzo T., D’Ausilio A. (2015). Generalization of motor resonance during the observation of hand, mouth, and eye movements. J. Neurophysiol..

[24] Güldenpenning I., Wilfried K., Matthias W. (2017). How to Trick Your Opponent: A Review Article on Deceptive Actions in Interactive Sports. Front. Psychol..

[25] Urgesi C., Savonitto M.M., Fabbro F., Aglioti S.M. (2012). Long- and short-term plastic modeling of action prediction abilities in volleyball. Psychol. Res..

[26] Anderson D.N., Gottwald V.M., Lawrence G.P. (2019). Representational Momentum in the Expertise Context: Support for the Theory of Event Coding as an Explanation for Action Anticipation. Front. Psychol..

